# Seasonal mortality trends for hospitalised patients with acute kidney injury across England

**DOI:** 10.1186/s12882-023-03094-5

**Published:** 2023-05-24

**Authors:** Esther Wong, Javeria Peracha, David Pitcher, Anna Casula, Retha Steenkamp, James F Medcalf, Dorothea Nitsch

**Affiliations:** 1 Renal Registry, Kidney Association, Brandon House 20a1, Southmead Road, Bristol, BS34 7RR UK; 2 Population Health Sciences, Bristol Medical School, University of Bristol, Bristol, UK; 3 Department of Renal Medicine, Royal Wolverhampton NHS Trust, Wolverhampton, WV10 0QP UK; 4 Department of Renal Medicine, University College London, London, UK; 5 Department of Health Sciences, University of Leicester, Leicester, LE1 7RH UK; 6 John Walls Renal Unit, University Hospitals Leicester NHS Trust, Leicester, LE1 5WW UK; 7 Faculty of Epidemiology and Population Health, London School of Hygiene and Tropical Medicine, London, WC1E 7HT UK; 8 Department of Nephrology, Royal Free London NHS Foundation trust, London, NW3 2QG UK

**Keywords:** Acute kidney Injury, Seasonality, Mortality risk, Centre-Variation

## Abstract

**Background:**

Incidence of acute kidney injury (AKI) is known to peak in winter months. This is likely influenced by seasonality of commonly associated acute illnesses. We set out to assess seasonal mortality trends for patients who develop AKI across the English National Health Service (NHS) and to better understand associations with patient ‘case-mix’.

**Methods:**

The study cohort included all hospitalised adult patients in England who triggered a biochemical AKI alert in 2017. We modelled the impact of season on 30-day mortality using multivariable logistic regression; adjusting for age, sex, ethnicity, index of multiple deprivation (IMD), primary diagnosis, comorbidity (RCCI), elective/emergency admission, peak AKI stage and community/hospital acquired AKI. Seasonal odds ratios for AKI mortality were then calculated and compared across individual NHS hospital trusts.

**Results:**

The crude 30-day mortality for hospitalised AKI patients was 33% higher in winter compared to summer. Case-mix adjustment for a wide range of clinical and demographic factors did not fully explain excess winter mortality. The adjusted odds ratio of patients dying in winter vs. summer was 1.25 (1.22–1.29), this was higher than for Autumn and Spring vs. Summer, 1.09 (1.06–1.12) and 1.07 (1.04–1.11) respectively and varied across different NHS trusts (9 out of 90 centres outliers).

**Conclusion:**

We have demonstrated an excess winter mortality risk for hospitalised patients with AKI across the English NHS, which could not be fully explained by seasonal variation in patient case-mix. Whilst the explanation for worse winter outcomes is not clear, unaccounted differences including ‘winter-pressures’ merit further investigation.

**Supplementary Information:**

The online version contains supplementary material available at 10.1186/s12882-023-03094-5.

## Introduction

Acute Kidney Injury (AKI) is a clinical syndrome frequently observed amongst hospitalised patients. [1–5] It is characterised by a sudden reduction in kidney function that can occur in association with a wide range of common illnesses, including sepsis, respiratory tract infections, congestive cardiac failure, stroke, trauma and following major surgery [4]. Over 500,000 cases of AKI were recorded in England during 2018 alone. [6] Patients who develop AKI are vulnerable to serious complications including prolonged hospitalisations, readmissions, kidney failure requiring dialysis, progression to chronic kidney disease, increased cardiovascular risk and death.[2, 5, 7−9] Reported mortality rates for patients with AKI are high, ranging between 15 and 30%, dependent on the definition of AKI used, clinical setting (community or hospital-acquired AKI) and AKI severity. [10,11] AKI was first highlighted as a significant patient safety concern across the English National Health Service (NHS) following the National Confidential Enquiry into Patient Outcomes and Death (NCEPOD) report in 2009, which suggested that many cases of AKI and associated adverse outcomes could have been avoided through improvements in patient care. [10].

Previous studies from Wales and Japan, where there is a temperate climate, have noted an increase in AKI incidence and mortality rates for patients who develop AKI during winter months. [12,13] The reasons behind this are not fully understood but have been attributed in part to the well described “excess winter mortality” of illnesses commonly associated with AKI e.g. ischaemic heart disease, heart failure and respiratory conditions.

In this study we set out to examine, for the first time, seasonal variation in mortality rates for patients who develop AKI across the English NHS, using a national database of biochemically detected AKI cases held at the UK renal registry (UKRR) linked to NHS hospitals administrative data (Hospital Episodes Statistics, HES) and mortality feeds from the Office for National Statistics (ONS). Case-mix adjustment for a wide-range of demographic and clinical variables has been undertaken to try and better understand their association with observed seasonal mortality trends. Centre-variation in these values has also been explored to improve our understanding of the differential impact of season on AKI patient mortality across NHS Trusts.

## Materials and methods

### Study designs

This was a retrospective observational study of adult hospitalised patients (> 18 years) with AKI in England, between 01/01/2017–31/12/2017, at acute healthcare organisations (termed ‘trusts’ in the NHS). Each NHS Trust may consist of between one and five acute hospitals, with some shared infrastructure e.g. laboratory information management systems (LIMs).

### Dataset

Laboratories in England are mandated to monitor for significant elevations in serum creatinine using the national AKI alerting algorithm published by NHS England. [14] AKI alerts for AKI stages 1, 2 and 3 (graduated by level of severity) are sent by laboratories to the treating clinicians and also the UKRR. This database of laboratory returned biochemical AKI alerts held at the UKRR is known as the AKI Master Patient Index (MPI).

AKI episodes and clinical settings are defined as per the 2020 UKRR AKI report. [6] The date of a first AKI episode is defined as the date of the first AKI alert received by the UKRR for each individual patient. Subsequent alerts are only considered to be a further episode of AKI if at least 30 days have passed since the last alert. If an episode appears to last more than 90 days, duration of the episode is truncated to day 90 to align with the KDIGO definition of chronicity after 90 days of an AKI episode. [6,15]

### Study cohort

The UKRR routinely link data for patients in the AKI-MPI to NHS Hospital Episode Statistics (HES) and mortality feeds from the Office for National Statistics (ONS). In this analysis, we studied patients in the dataset between 01/01/2017–31/12/2017. Appendix A1a and A1b outlines further details on cohort selection.

### Exposure

Seasons were defined meteorologically as spring (March, April, May), summer (June, July, August), autumn (September, October, November) and winter (December, January, February).

### Outcome

The main outcome of interest was mortality in hospital or within 30 days of discharge.

### Covariates

Clinical variables included in our model were primary diagnosis at hospital admission (see appendix A2), comorbidity RCCI (AKI specific re-weighting of Charlson Comorbidity Index as detailed in Appendix table A1c ), age, sex, admission method, peak AKI stage, index of multiple deprivation quintiles (IMD) derived from patient postcode, ethnicity, whether AKI was community acquired prior to hospital admission (CAH) or hospital acquired (HA). [16].

### Statistical modelling

All analyses were conducted using SAS 9.4. Proportions of patients with different clinical characteristics across the seasons were presented. Chi-square tests were performed to see if patient characteristics have an association with season. P values less than 0.05 were considered statistically significant.

Multivariable logistic regression was used to assess whether season is associated with the 30-day mortality, adjusting for clinical variables. This paper aims to demonstrate the net effect of season to mortality of AKI patients. Since we are not focused on the mortality prediction, or interpreting each of the covariate effects on mortality, we have not shown the individual point estimates for all the covariates. We did not model any interactions as we were concerned about overfitting models and spurious associations. Age was found to act as a non-linear covariate and was therefore included as a non-linear cubic spline (Appendix A1d). Risk factors were entered into the model in each forward step sequentially, allowing assessment of the impact of each risk factor on 30-day mortality and whether they were confounded by other risk factors already in the model. The model was internally validated using Hosmer and Lemeshow measure of concordance. The concordance statistic of the model was 77%, indicating acceptable goodness of fit.

Season was also modelled by centre to explore how seasonal mortality trends varied across NHS Trusts. The ‘Akaike Information Criterion (AIC)’ was used for variable selection and for assessing the inclusion of interactions between season and centre. [17].

## Results

### Seasonal Variation of patient characteristics for hospitalised patients with AKI

**Fig. 1:**
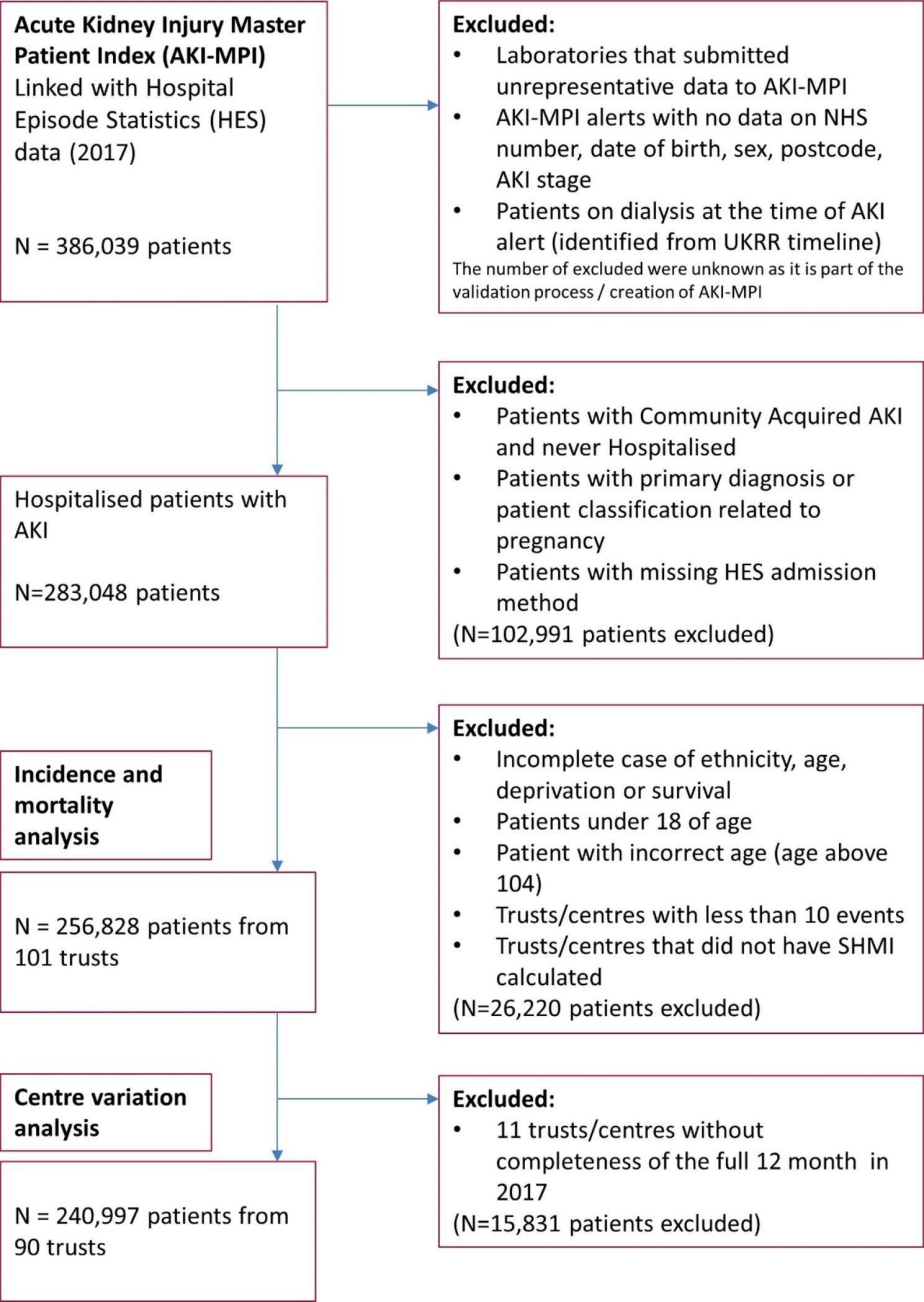
CONSORT diagram for the cohort analysis

There were 283,048 hospitalised patients with a biochemical AKI alert included in our final cohort (Fig. 1). 68,731 AKI episodes occurred in winter (December to February), which was 11% higher than the number of AKI episodes in summer (June to August), when there was 61,703.

Patients who developed AKI in winter were more likely to be older, to have had an emergency admission, higher RCCI and to have reached peak AKI stage 3 (Table 1). Seasonal trends in AKI alert numbers varied with age: in age group 18–39, 25.9% of AKI episodes occurred in summer and 23.8% in winter, whereas in the age group > 75, 23.4% of AKI episodes occurred in summer and 27.9% in winter.

**Table 1:** Demographics and clinical characteristics of hospitalised AKI patients in England by season in 2017

			Spring	Summer	Autumn	Winter	P-value
**Total number of patients**	**N (row%)**		64,247 (25%)	61,703 (24%)	62,147 (24.2%)	68,731 (26.8%)	
**Median age**	**N (IQR)**		76.8 (65.3–85.1)	76.2 (64.2–84.7)	76.3 (64.5–85)	77.7 (66.4–85.8)	
**Age group**							p < 0.001
	**18–39**		2,991 (24.4%)	3,182 (25.9%)	3,180 (25.9%)	2,923 (23.8%)	
	**40–64**		12,757 (24.9%)	12,890 (25.2%)	12,761 (24.9%)	12,791 (25%)	
	**65–75**		13,504 (25.2%)	12,953 (24.2%)	13,048 (24.4%)	14,044 (26.2%)	
	**> 75**		34,995 (25%)	32,678 (23.4%)	33,158 (23.7%)	38,973 (27.9%)	
**Reweighted Charlson Comorbidity Index group (RCCI)**	**No comorbidities**		27,309 (25.4%)	26,166 (24.3%)	25,993 (24.2%)	28,129 (26.1%)	p < 0.001
	**CCI = 1**		14,225 (24.7%)	13,596 (23.6%)	13,967 (24.2%)	15,822 (27.5%)	
	**CCI = 2**		10,354 (24.8%)	9847 (23.6%)	9992 (23.9%)	11,529 (27.6%)	
	**CCI > 2**		12,359 (24.8%)	12,094 (24.2%)	12,195 (24.4%)	13,251 (26.6%)	
**Emergency/Elective admission**	**Elective**		5,922 (24.6%)	6,147 (25.5%)	6,541 (27.1%)	5,488 (22.8%)	p < 0.001
	**Emergency**		58,325 (25.1%)	55,556 (23.9%)	55,606 (23.9%)	63,243 (27.2%)	
**Peak AKI stage**	**1**		41,838 (25%)	40,248 (24.1%)	41,130 (24.6%)	44,102 (26.4%)	p < 0.001
	**2**		12,617 (24.9%)	12,266 (24.2%)	11,999 (23.7%)	13,821 (27.3%)	
	**3**		9,792 (25.2%)	9,189 (23.7%)	9,018 (23.2%)	10,808 (27.9%)	
**Deprivation quintile (IMD)**	**Least deprived 1**		10,464 (24.8%)	10,273 (24.4%)	10,295 (24.4%)	11,140 (26.4%)	p = 0.11
	**2**		12,138 (24.9%)	11,715 (24.1%)	11,658 (23.9%)	13,185 (27.1%)	
	**3**		13,387 (25.5%)	12,476 (23.7%)	12,629 (24%)	14,109 (26.8%)	
	**4**		13,439 (24.9%)	13,023 (24.1%)	13,136 (24.4%)	14,338 (26.6%)	
	**Most deprived 5**		14,819 (24.9%)	14,216 (23.9%)	14,429 (24.3%)	15,959 (26.9%)	
**Sex**	**F**		31,819 (24.9%)	30,663 (24%)	30,725 (24.1%)	34,383 (26.9%)	p = 0.15
	**M**		32,428 (25.1%)	31,040 (24%)	31,422 (24.3%)	34,348 (26.6%)	
**Community acquired hospitalised (CAH)/Hospital acquired (HA)**	**CAH**		35,381 (25%)	34,491 (24.4%)	33,974 (24%)	37,756 (26.7%)	p < 0.001
	**HA**		28,866 (25.1%)	27,212 (23.6%)	28,173 (24.5%)	30,975 (26.9%)	

### Seasonal variation in mortality for patients who developed AKI

**Fig. 2:**
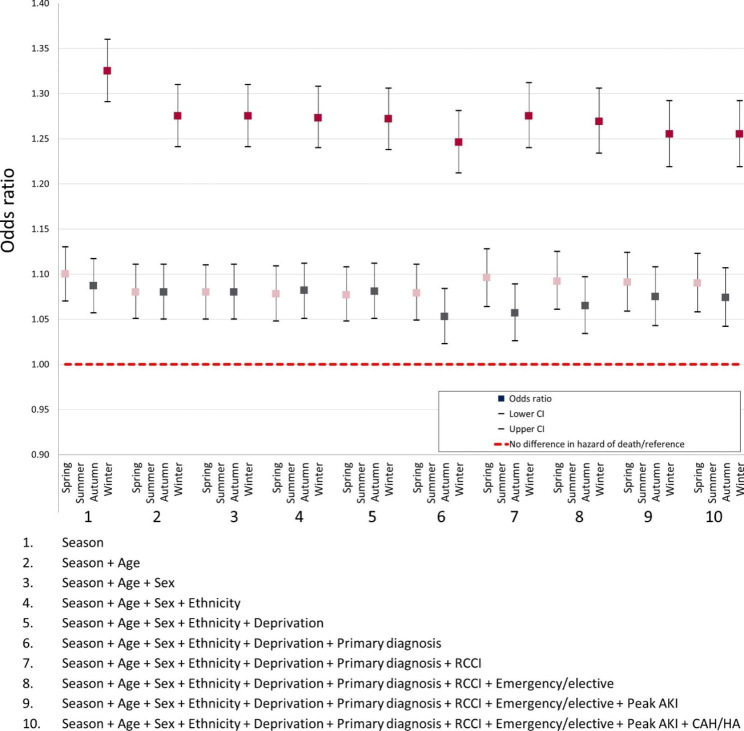
30-day mortality odds ratio by season. This figure demonstrates the impact on 30-day mortality for patients in our cohort after sequential adjustments for covariates including comorbidity RCCI, primary diagnosis, age, admission method, peak AKI stage, index of multiple deprivation quintiles (IMD), ethnicity, if AKI was community acquired then hospitalised (CAH) or hospital acquired (HA) and sex, with summer season as reference group

Crude 30-day mortality was 33% higher for patients who had an episode of AKI in winter compared to those patients who developed AKI in summer [OR 1.33 (1.30–1.36), p < 0.01] (Fig. 2). Comparing ‘model 1’ which included season only, and ‘models 2–5’, which included sequential adjustments for demographic factors including age, sex, ethnicity and deprivation (IMD) - the odds ratio of mortality in winter appeared to be most influenced by inclusion of patient age. Further models demonstrated a fall and then rise in mortality odds ratios when adjusting for primary diagnosis group and RCCI respectively. Odds ratio of mortality in winter months then fell again slightly after adjustments for type of admission (emergency vs. elective), peak AKI stage and type of AKI (community vs. hospital acquired). In the fully adjusted model, odds ratio for mortality in winter remained significantly higher (25%) than in summer [OR 1.26 (1.22–1.29), p < 0.01].

### Seasonal variation in mortality within specific primary diagnosis groups

The most common primary diagnosis group at admission for hospitalised AKI patients in our cohort were respiratory infections (RTI) (N = 31,976, 12.5%) and cardiovascular diseases (CVD) (N = 24,275, 9.5%), Table 2. The third most common diagnosis was sepsis, however due to changes in coding of sepsis during the time period of our study we were unable to analyse this data further (*for details see Appendix A3).* Other common diagnoses included gastrointestinal conditions (N = 19,931, 7.8%), malignancy (N = 19,365, 7.5%), urinary tract infections (UTI) (N = 11,052, 4.3%)) and hip fractures (N = 6,805, 2.6%). There was clear evidence of seasonal variation in the number of patients in our cohort who had these primary diagnoses.

**Table 2:** Percentage of hospitalised AKI patients in 2017 by Primary disease group across the seasons

Primary Disease group*			Spring	Summer	Autumn	Winter	Chi-Sq p-value
	N	Col %	Row %	Row %	Row %	Row %	
Respiratory Infections	31,976	12.50	23.61	17.12	20.59	38.68	p < 0.001
Cardiovascular Disease	24,275	9.45	25.35	23.40	25.18	26.07	p < 0.001
Sepsis**	23,396	9.11	24.31	27.48	28.86	19.35	p < 0.001
Gastrointestinal conditions	19,931	7.76	25.18	25.69	23.70	25.42	p < 0.001
Malignancy	19,365	7.54	24.02	25.49	26.24	24.24	p < 0.001
Urinary Tract Infections	11,052	4.30	25.66	21.63	20.45	32.27	p < 0.001
Hip fractures	6,805	2.65	25.33	22.29	24.54	27.83	p < 0.001
Cerebrovascular disease	4,817	1.88	24.35	23.35	25.70	26.59	p = 0.008
Other	115,211	44.9	25.56	25.20	24.06	25.17	p < 0.001

*see Appendix A2 **see Appendix A3

**Fig. 3:**
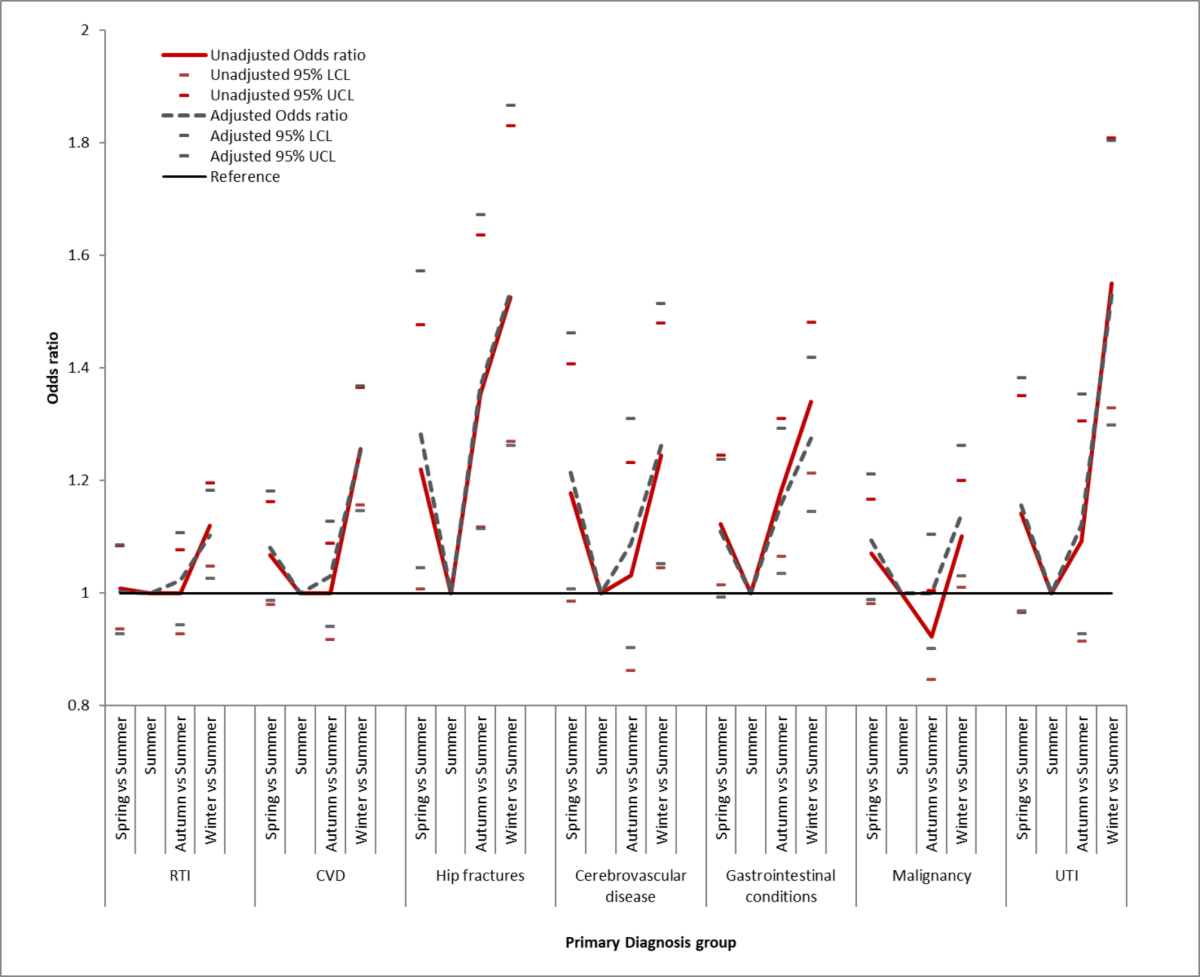
Odds ratio for unadjusted and adjusted 30-day mortality of AKI patients with RTI, CVD and Hip fractures, Cardiovascular Disease, Gastrointestinal conditions, Malignancy and UTI separately as primary diagnosis. Unadjusted 30-day mortality is shown alongside adjusted 30-day mortality (with age, sex, ethnicity, deprivation, RCCI, admission method, peak AKI stage, and hospital vs. community acquired included in the model)

Figure 3 illustrates the odds ratios for mortality by season for patients with AKI grouped by primary diagnosis group except for sepsis. In the fully adjusted model, variation in mortality risk across seasons was most pronounced for patients with a primary diagnosis of hip fractures and UTI.

### Centre variation in seasonal mortality odds ratios

**Fig. 4:**
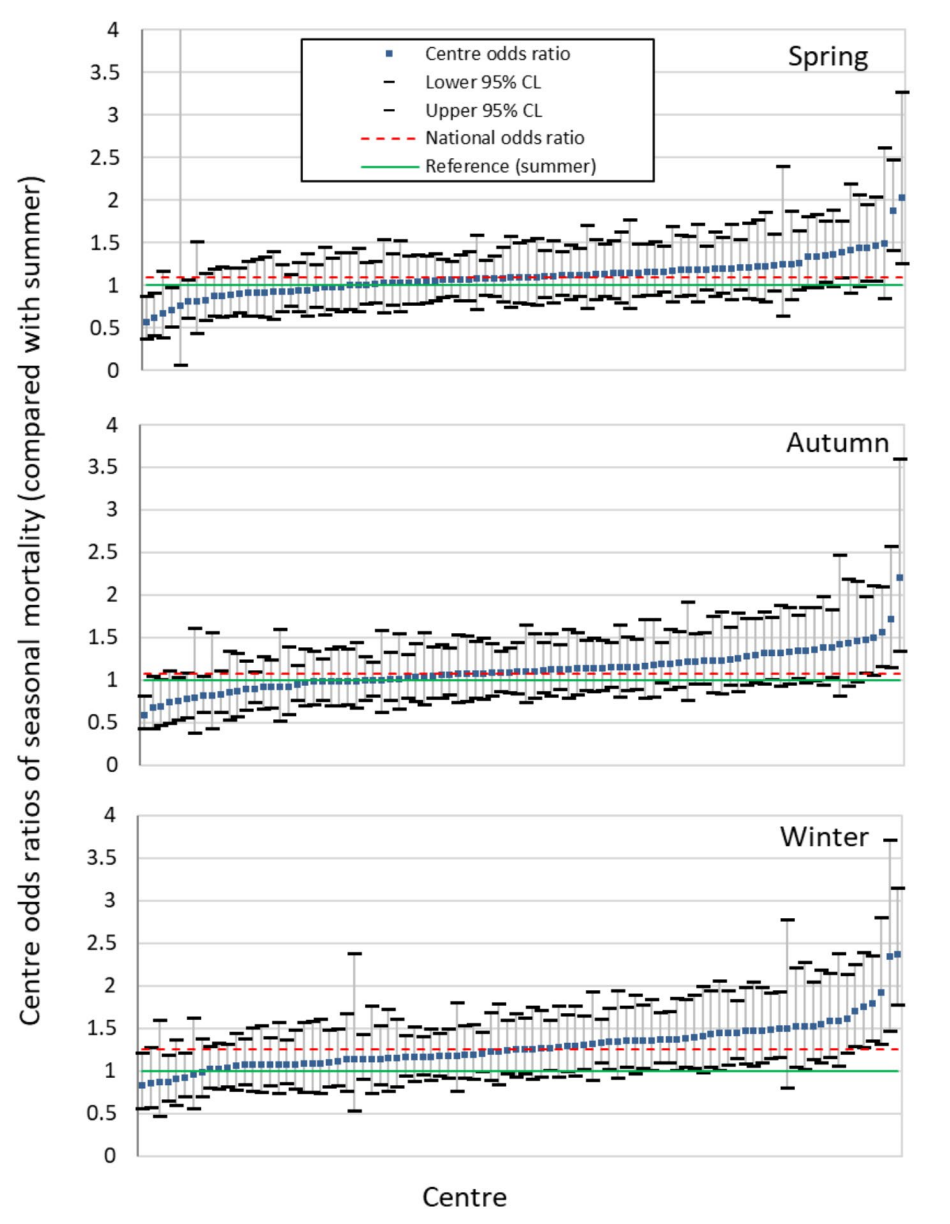
Odds ratio of 30-day mortality following AKI alert by centre, by season (adjusting for age, sex, ethnicity, deprivation, primary diagnosis, RCCI, elective/emergency admission, peak AKI stage, and community/hospital acquired AKI. Note only centres with 12 months of complete data have been included in this analysis)

To model the centre-variation on 30-day mortality across seasons, the best fitting model of centre was a fixed effect model where, after taking account of case-mix and demographics of patients with AKI, mortality is different between hospitals (ranging from 16.1 to 25.1%) (see Appendix A4). Figure 4 shows that the fully adjusted national odds ratio (red dotted lines) for patient mortality in winter (OR = 1.25 [1.22–1.29]) was higher than that in spring (OR = 1.09 [1.06–1.12]) or autumn (OR = 1.07 [1.04–1.11]), when comparing to summer as reference (green solid line). 35 out of 90 individual centres had a mean winter vs. summer mortality odds ratios (including lower 95% CL) that was higher than 1. When examining national variation in winter vs. summer mortality odds ratios, 9 out of 90 (10%) centres were outliers, with mean 30-day mortality odds ratios (including CI) at 6 centres lying above the national average and at 3 centres below it. Similarly when exploring autumn vs. summer mortality odds ratios, 9 out of 90 centres were outliers (4 above and 5 below the national average) and in spring vs. summer there were 7 out of 90 centres that were outliers (3 above and 4 below the national average).

## Discussion

Our analysis of a large national cohort of hospitalised patients with biochemically defined AKI across England confirmed that the incidence of AKI was seasonal, peaking in winter months. Unadjusted 30-day mortality for patients who developed AKI was 33% higher in winter compared to summer, and this pattern was repeated across a wide range of primary diagnosis groups, persisting after extensive adjustments for patient case-mix, including socio-demographic and clinical risk factors. The impact of season varied somewhat across NHS trusts in our cohort, with evidence of clear outlier centres.

Our main study findings are in keeping with previously reported studies in this area. This includes an analysis of a large Japanese cohort of 81,279 patients with biochemically defined AKI from across 38 hospitals, where AKI incidence and mortality was found to be significantly higher in winter months. [12] Another national study of 48,457 patients with biochemical AKI alerts from Wales similarly found that the incidence of AKI fell throughout the four quarters of the calendar year (highest between January – March) with mortality reported as highest for patients who developed AKI in winter months. [13] Our analysis includes a significantly larger patient cohort than these previous studies, with 283,048 AKI episodes studied in total, across 90 NHS Trusts. Our best-fitting statistical model implies that every centre goes into the winter season with a different AKI baseline mortality, with then a uniform additional 30-day mortality risk after AKI in the winter that is not explained by demographic or case-mix features. Exploration of how seasonal mortality trends vary across different centres is designed to gain insight into unwarranted variation in patient outcomes in a universal health care system (NHS), something that has not previously been investigated.

Measurement of AKI incidence and outcomes has frequently been described as a valuable patient safety barometer for NHS hospitals. [18,19] AKI is observed across a wide range of hospital settings and ‘good’ AKI care is often closely associated with good overall patient care. We hypothesise that the observed excess winter mortality risk for patients who developed AKI in our cohort (persisting despite case-mix adjustment) may potentially reflect “winter-pressures”. This is a phenomenon of increasing concern across the NHS. As temperatures fall, increased demand for medical care, changes in how patients access healthcare, and how services manage the flow of patients, all contribute to deterioration in the performance of healthcare providers from January onwards. [20,21] The clinical care pathways, resources and resilience for NHS hospitals are not adjusted for in our model and they may be varied under winter pressures. Trusts that are identified as high outliers in our analysis may benefit from further review to better understand and address factors that may be contributing to excess winter mortality risk for their AKI patients. Whereas for trusts without excess winter AKI mortality, we assume they had good service management that allowed for the increase in demand of medical care in the winter and adjust their capacity accordingly. They could share their experience and best practice in order to improve performance of less well performing trusts.

Patients who had a primary diagnosis of UTI in our cohort were most vulnerable to excess winter mortality risk (OR = 1.55[1.30–1.80]), alongside patients with a primary diagnosis of hip fractures (OR = 1.53[1.27–1.83]). Both of these diagnoses are commonly associated with increased patient frailty. Although our statistical model adjusts for age and co-morbid conditions, it is difficult to fully account for the severity of these conditions and the concept of frailty using administrative data alone. Similarly we cannot account for reverse causality in our models, where patients are already dying at the time that they trigger an AKI alert. Nonetheless, frail patients are likely to be most vulnerable to “winter pressures” on the healthcare system and this finding merits further focussed investigation to see if outcomes can be improved for these patient groups. Measures such as vaccination programmes against seasonal influenza and pneumococcus, hospital acute frailty units and specialist “orthogeriatric” teams are already widely utilised across the English NHS [22–25].

AKI mortality was most influenced by comorbidity, which we have adjusted for with the RCCI (Supplementary file section A1c Table A1). A limitation of this study is that there might be other unadjusted risk factors such as having interventions associated with AKI risk such as cardiovascular surgery or type of speciality treatment, including ICU stay, however, these variables are to some extent indirectly captured by the RCCI, type of admission, primary disease group, and peak AKI severity which were adjusted for in our analysis.

In this analysis we have restricted our cohort to hospitalised patients with AKI, as clinical variables for case-mix adjustment had to be derived from HES. We therefore could not study seasonal outcomes for patients who developed AKI in the community and were not hospitalised (approximately 30% of all AKI cases in England). [6] it is difficult to comment on the potential impact of “selection bias” on our findings, due to variability in thresholds for admission for patients with AKI across different seasons. We were also unable to capture a complete national cohort of patients with hospitalised AKI. This was due to incomplete data returns from laboratories to the UKRR during the study time period.

## Conclusion

In this study we have demonstrated that the incidence of AKI peaks in winter months across the English NHS. Mortality risk for hospitalised patients with AKI is also higher in winter, despite accounting for a range of clinical variables including patient demographics, primary diagnosis, comorbidities and AKI severity. This unexplained variation, including the possible impact of “winter-pressures” on outcomes for patients with AKI merits further investigation.

## Electronic supplementary material

Below is the link to the electronic supplementary material.

Supplementary Material 1

## Data Availability

The data that support the findings of this study are available from the UK Renal Registry but restrictions apply to the availability of these data, which were used under license for the current study, and so are not publicly available. Data are however available from the authors upon reasonable request and with permission of the UK Renal Registry – Contact Esther Wong at ukrr-research@renalregistry.nhs.uk.

## Abbreviations

AIC: Akaike Information Criterion
AKI: Acute Kidney Injury
AKI1: Acute Kidney Injury Stage 1
AKI2: Acute Kidney Injury Stage 2
AKI3: Acute Kidney Injury Stage 3
CCI: Charlson Comorbidity Index
CAH: Community acquired, subsequently hospitalised
CI: Confidence Interval
CL: Confidence Limit
CVD: Cardiovascular Disease
HA: Hospital acquired
H-AKI: Post-Hospitalisation Acute Kidney Injury
HES: Hospital Episodes Statistics
IMD: Index of Multiple Deprivation
KDIGO: Kidney Disease:Improving Global Outcomes
MPI: Master Patient Index
NCEPOD: National Confidential Enquiry into Patient Outcomes and Death
NHS: National Health Service
ONS: Office for National Statistics
RRT: Renal Replacement Therapy
RTI: Respiratory Infections
RCCI: Re-weighted Charlson Comorbidity Index
SCr: Serum Creatinine
SHMI: Summary Hospital Mortality Indicator
SMR: Standardised Mortality Ratio
UKRR: UK Renal Registry
UTI: Urinary Tract Infections
VPC: Variance Partition Co-efficient
