# Supplementary material for: Seasonal mortality trends for hospitalised patients with acute kidney injury across England
Source: BMC Nephrol. 2023 May 24;24:144. doi: 10.1186/s12882-023-03094-5 (PMC10210343; doi:10.1186/s12882-023-03094-5)
Supplement: Supplementary file 1 — Supplementary Material 1 [file 12882_2023_3094_MOESM1_ESM.docx]

# Supplementary information

# Appendix

## Appendix A1 Cohort and methods

### A1a Details of inclusion and exclusion and clinical setting

We have excluded mother and baby using delivery facilities only, day case, regular attendances, pre/post-partum admission, births admission, transfer of any admitted patient from another hospital provider other than in an emergency and unknown admission.

In this paper, we have focused on patients who had an AKI episode in 2017, and eventually hospitalised during 2017 at one point. Patients are divided into two groups: Community acquired (CA), subsequently hospitalised (CAH) AKI: AKI episode started before an inpatient admission or in the first 2 days of an inpatient admission; and Hospital acquired (HA) AKI: AKI episode had started from the 3^rd^ day of an inpatient admission onwards.

The AKI-MPI also captured patients who had an AKI episode but no inpatient admission during the AKI episode in 2017, in other words they are CA AKI. This group of patients could not be complimented with the same level of clinical information by HES data, therefore they are not included in our analyses. In the UKRR AKI report^6^, it has described this group of patient as younger, with lower peak AKI and included more females than expected.

### A1b Missing data

The completeness of the data for this cohort for most of the covariates are 100%, except for deprivation (postcode) with 0.3%, ethnicity with 4.9%, and age with <0.1% of missing data. We have also excluded 1.7% of patients who are aged < 18, as the reference creatinine in paediatric patients is very different from adult patients, and their risk to AKI is also different from adults. (https://www.thinkkidneys.nhs.uk/aki/wp-content/uploads/sites/2/2019/12/AKI-Guidance-paediatric-patients-Dec2019.pdf) Furthermore, this study has involved analysis by centre, so we would like to include centres that are more comparable, therefore we have also excluded patients from NHS foundation trust and centres with less than ten events of 30-day mortality, as well as trusts without published Summary Hospital-level Mortality Indicator (SHMI) reports on mortality.( Digital N. About the Summary Hospital-level Mortality Indicator (SHMI). Accessed July 2020, https://digital.nhs.uk/data-and-information/publications/ci-hub/summary-hospital-level-mortality-indicator-shmi) Overall, we have included 256,828 AKI patients, which is 90.7% of the initial cohort. In the centre variation analysis, we have further excluded the trusts without completeness of the full 12 months in 2017, to ensure we covered the same number of trusts throughout the year, which is 85.1% of the initial cohort.

### A1c Re-weighted Charlson comorbidity Index

**Table A1** Re-weighted Charlson comorbidity Index (RCCI) using all hospitalised AKI patients in England^11^

| Comorbidity | AKI Reweighted CCI |
| --- | --- |
| MI | 0 |
| Cerebrovascular disease | 1 |
| Heart Failure | 2 |
| Rheumatic disease | 0 |
| Dementia | 1 |
| Diabetes | 0 |
| Mild liver disease | 2 |
| Peptic ulcer disease | 1 |
| Peripheral vascular disease | 1 |
| Chronic pulmonary disease | 1 |
| Malignancy | 2 |
| Diabetes complications | 0 |
| Paraplegia | 1 |
| Renal disease | 0 |
| Malignancy metastatic solid tumour | 3 |
| Medium or severe Liver disease | 3 |

* Adjusted for age, sex, level of AKI alert, month of AKI alert, and each comorbidity group individually

### A1d Details of consideration on non-linearity of age

Age was tested in the model as both a linear and a non-linear term. A linear term assumes a relationship between age and the probability of death is constant as age increases, whereas a non-linear term allows the relationship to vary depending on the age. We used a natural cubic spline to explore non-linearity, allowing cubic expressions defined by “knots” at each quartile of age. The result of the age cubic spline model was plotted with mortality against the age, there was an obvious non-linearity at age between 60 and 80 years, therefore age was modelled as a non-linear cubic spline.

## Appendix A2 Primary disease group

Primary diagnosis group were determined from available HES data using the “summary hospital mortality indicator” (SHMI) methodology (Campbell, M.J., Jacques, R.M., Fotheringham, J., Maheswaran, R. and Nicholl, J., 2012. Developing a summary hospital mortality index: retrospective analysis in English hospitals over five years. BMJ, 344):

Patients in some of the SHMI diagnosis groups were combined together into larger categories by physicians to form broader categories that reflect patient groups commonly seen in routine clinical practice i.e. Respiratory Infections, Cardiovascular Disease, Sepsis, Gastrointestinal conditions, Malignancy, Urinary Tract Infections, Hip fractures and Cerebrovascular disease. These larger diagnosis groups encompassed all of the most common SHMI primary diagnosis groups observed for our cohort. The “other” category (diagnosis groups that were outside of Table A3), although large, consists of individual SHMI diagnosis groups that had a low number of patients that could not be grouped together to form larger unified categories. Grouping these diagnoses as one group allows us to control for the effect of the main conditions that have a large effect on AKI mortality whilst not losing too many degrees of freedom in our statistical model.

The primary disease group are congregated using to the SHMI diagnosis group in the HES data according to the following table:

**Table A3** Primary disease group definition

| **Primary disease group** | **HES - SHMI diagnosis group** |
| --- | --- |
| Cardiovascular Disease | Acute myocardial infarction |
|  | Coronary atherosclerosis and other heart disease |
|  | Other and ill-defined heart disease |
|  | Conduction disorders |
|  | Cardiac dysrhythmias |
|  | Cardiac arrest and ventricular fibrillation |
|  | Congestive heart failure; non hypertensive |
| Cerebrovascular disease | Acute cerebrovascular disease |
| Gastrointestinal conditions | Intestinal infection |
|  | Intestinal obstruction without hernia |
|  | Biliary tract disease |
|  | Liver disease; alcohol-related |
|  | Pancreatic disorders (not diabetes) |
|  | Gastrointestinal hemorrhage |
| Hip fractures | Fracture of neck of femur (hip) |
| Malignancy | Cancer of head and neck |
|  | Cancer of oesophagus |
|  | Cancer of stomach |
|  | Cancer of colon |
|  | Cancer of rectum and anus |
|  | Cancer of liver and intrahepatic bile duct |
|  | Cancer of pancreas |
|  | Cancer of other GI organs; peritoneum |
|  | Cancer of bronchus; lung |
|  | Cancer; other respiratory and intrathoracic |
|  | Melanomas, other cancer of skin |
|  | Cancer of breast |
|  | Cancer of uterus |
|  | Cancer of female genital organs |
|  | Cancer of ovary |
|  | Cancer of male reproductive organs |
|  | Cancer of bladder |
|  | Cancer of urinary organs |
|  | Cancer of brain and nervous system |
|  | Hodgkin's disease |
|  | Leukemias |
|  | Multiple myeloma |
|  | Other cancer (primary) |
|  | Secondary malignancies |
|  | Cancer of bone, thyroid and malignant neoplasm |
|  | Nutritional, endocrine and metabolic disorders |
|  | Non-Hodgkin's lymphoma |
| Respiratory Infections | Pneumonia (excluding TB/STD) |
|  | Acute bronchitis |
|  | COPD & bronchiectasis |
|  | Asthma |
|  | Aspiration pneumonitis; food/vomitus |
|  | Lung disease due to external agents |
|  | Upper respiratory disease, Diseases of mouth (non dental) |
| Sepsis | Septicaemia (except in labour), Shock |
|  |  |
| Urinary Tract Infections | Urinary tract infections |

## Appendix A3 Sepsis

Sepsis is well-known to be one of the leading causes of AKI. However we cannot explore AKI with sepsis as the primary diagnosis for our cohort, as there have been two recent noteworthy changes in the national guidelines of how sepsis should be coded in the HES data, the first in April 2017 and then again in April 2018 (<https://improvement.nhs.uk/documents/2472/Sepsis_FAQ.pdf>). Following the coding changes in April 2017, the practice of swift identification and treatment of a local infection early in its presentation resulted in an increase in the number of spells coded with a primary diagnosis of sepsis (details are in the reference document). Therefore we decided not to explore the sepsis data further.

## Appendix A4 Centre effect

**Table A4** Comparing Akaike Information Criterion (AIC) with different assumptions on centre effect

| **Variables in the model** | **AIC** |
| --- | --- |
| Final model | 226751.0 |
| **Final model + fixed centre effect** | **226161.4** |
| Final model + fixed centre effect + fixed centre*season interaction | 226345.8 |
| Final model + random centre intercept | 226290.5 |
| Final model + random centre*season slope | 226420.9 |
| Final model + random centre intercept + random centre*season slope | 226274.8 |

Final model has included the following variables: season, comorbidity (reweighted Charlson Comorbidity Score), primary diagnosis, age, admission method, peak AKI stage, index of multiple deprivation quintiles (IMD), ethnicity, if AKI was community acquired then hospitalised (CAH) or hospital acquired (HA) and sex.

**Table A4** shows different models of centre effect with different assumptions and compare their Akaike Information Criterion (AIC), which is an estimator of out-of-sample prediction error and thereby relative quality of statistical models for a given set of data, with lower the AIC highlighting a better quality of the model. Modelling centre as a single fixed effect is an easy way to demonstrate the seasonal effect by centre, moreover it fits best with the lowest AIC, followed by a random centre intercept being the second best choice. Adding the fixed centre and season interaction term did not improve the model quality, it means although different centres had differing patient mortality, the overall seasonal effect was not different across centres. However we are still interested in visualizing the seasonal effect by the individual centre, to see if there were outliers, therefore we continued to compare them in Figure 4.
